# Stereotactic radiosurgery for single brain metastases from non-small cell lung cancer: progression of extracranial disease correlates with distant intracranial failure

**DOI:** 10.1186/1748-717X-8-64

**Published:** 2013-03-19

**Authors:** Marie-Adele S Kress, Eric Oermann, Matthew G Ewend, Riane B Hoffman, Huma Chaudhry, Brian Collins

**Affiliations:** 1 Department of Radiation Medicine, Lombardi Comprehensive Cancer Center, Georgetown University Hospital, Lower Level Bles, 3800 Reservoir Road, N.W, Washington, DC 20007, USA; 2 Department of Neurosurgery, University of North Carolina: Chapel Hill, 170 Manning Drive, CB 7060, Chapel Hill, NC 27599, USA; 3 George Washington University, Ross Hall, 2300 Eye Street,NW, Washington DC 20037, USA

**Keywords:** Brain metastases, Non-small cell lung cancer, Palliative care, Distant intracranial failure, Stereotactic radiosurgery, CyberKnife

## Abstract

**Background:**

Limited data exist regarding management of patients with a single brain lesion with extracranial disease due to non-small cell lung cancer (NSCLC).

**Methods:**

Eighty-eight consecutive patients with a single brain lesion from NSCLC in the presence of extracranial disease were treated with stereotactic radiosurgery (SRS) alone. Local control (LC), distant intracranial failure (DIF), overall survival (OS), and toxicity were assessed. The logrank test was used to identify prognostic variables.

**Results:**

Median OS was 10.6 months. One-year DIF was 61%; LC 89%. Treatments were delivered in 1-5 fractions to median BED_10_ = 60Gy. Five patients developed radionecrosis. Factors associated with shortened OS included poor performance status (PS) (p = 0.0002) and higher Recursive Partitioning Analysis class (p = 0.017). For patients with PS 0, median survival was 22 months. DIF was associated with systemic disease status (progressive vs. stable) (p = 0.0001), as was BED (p = 0.021) on univariate analysis, but only systemic disease (p = 0.0008) on multivariate analysis.

**Conclusions:**

This study identifies a patient population that may have durable intracranial control after treatment with SRS alone. These data support the need for prospective studies to optimize patient selection for up-front SRS and to characterize the impact of DIF on patients’ quality of life.

## Background

Brain metastases are common, occurring in 20-40% of cancer patients and contributing to 20% of annual cancer deaths [1]. Brain metastases are particularly common among patients with non-small cell lung cancer (NSCLC), even at the time of diagnosis, accounting for approximately 18-64% of all brain metastasis diagnoses [2]. However, some of these patients will present with limited intracranial disease, with one or few metastatic lesions. Overall prognosis of patients with brain metastases is limited, but has been shown to vary significantly, based on factors such as tumor histology, number of lesions, patient age and performance status [3-7].

With advances in systemic therapy, tailoring intracranial radiation therapy (RT) to individual patients’ clinical circumstances is increasingly important. Recent studies have evaluated the role of stereotactic radiosurgery (SRS) in combination with or instead of surgery and/or whole brain radiation therapy (WBRT) in carefully selected patient subgroups [8-10]. The goal of using SRS as a stand-alone therapy has been to maximize local control (LC) while minimizing toxicities and adverse impact on quality of life (QOL) [11-14]. However, some patients still benefit from up-front WBRT as part of their treatment, as demonstrated in Patchell’s study where 24% of patients treated with surgery alone had distant intracranial recurrence only, compared to 8% who received adjuvant WBRT [14]. Guidelines exist, but remain vague, regarding optimal treatment regimens for patients with metastatic NSCLC; as a result, SRS use remains heterogeneous [15-19]. Overall, significant limitations still exist regarding optimizing patient selection for up-front SRS as a single modality, since these patients present with heterogeneous clinical circumstances.

Historically, a limited cohort of patients with a solitary brain lesion due to metastatic NSCLC have been treated with surgery to achieve LC and ultimately long-term intracranial disease-free survival [20,21]. However, in patients with single brain metastases in the presence of active extracranial disease, LC, distant intracranial failure (DIF) and survival are less while characterized. This patient population is important, since brain metastases are often diagnosed in the presence of extracranial disease. Both LC and DIF can be significant to patients’ long-term prognosis as well as their functional status and QOL, making the decision of which up-front RT technique(s) to select particularly important.

To better define prognosis for patients with a single brain metastases from NSCLC and more effectively characterize which patients are at relatively high or low risk for distant intracranial recurrence, retrospective data from two institutions were pooled for evaluation of clinical outcomes and toxicity, as well as assessment of clinical prognostic variables.

## Methods

### Patient selection

This retrospective study was approved by the Institutional Review Boards of both Georgetown University and the University of North Carolina (UNC). Eighty-eight patients were identified who were treated between 2002 and 2011, 40 of whom were treated at UNC, and 48 of whom were treated at Georgetown. All patients had a pathologically confirmed diagnosis of non-small cell lung cancer. Patients were included with any stage NSCLC at initial diagnosis, but at the time of presentation with brain metastases they had to have documented extracranial disease that was either stable or progressing. Patients were included if they had a single brain lesion, as confirmed by magnetic resonance imaging (MRI). Those with more than one intracranial lesion considered suspicious for metastasis, as documented by MRI, were excluded. Patients’ performance statuses were estimated both using the Karnofsky Performance Status (KPS) and Eastern Cooperative Oncology Group (ECOG) scales, to allow for analysis by overall performance status (PS), Recursive Partitioning Analysis (RPA) classes and Diagnosis-Specific Graded Prognostic Index (DS-GPA) groups. Thirteen patients without radiographic follow-up were included in the analysis for overall survival (OS), but not for LC or DIF. One patient without any follow-up was not included in evaluation of OS, LC, or DIF.

### SBRT planning and treatment

Each patient underwent simulation in the supine position with creation of a custom immobilization device. A treatment planning computed tomography (CT) scan with slices of 1-3 mm thickness was used for treatment of all patients. The majority of patients also had contrast-enhanced, thin-slice MRI fused with the CT scan for treatment planning; the most commonly used treatment planning sequence was magnetization-prepared rapid acquisition with gradient echo (MPRAGE), which is a high-resolution T1 sequence with contrast. Contrast-enhanced CT scan was used at the discretion of the treating physician.

Gross tumor volume (GTV) was delineated on CT scan(s) and MRI (when applicable) after fusion in the MultiPlan treatment planning software. Typically, a circumferential margin of 1-3 mm was added to the GTV to create the clinical target volume (CTV), with no additional expansion to form the planning target volume (PTV). Adjacent critical structures were delineated as indicated, depending on the location of the treated tumor.

All treatments were performed using the CyberKnife system (Accuray, SunnyVale, CA) and were planned using Multiplan treatment software. This method uses an inverse-planning technique to generate conformal treatment plans with avoidance of critical structures. All treatments were delivered using 6 MV photons and were prescribed to an isodose line that provided adequate (>95%) coverage of the PTV. All treatments were performed using Xsight cranial tracking. Biologic equivalent dose (BED) was calculated for each fractionation scheme, with α/β ratio assumed to be 10, according to the following formula: BED_10_ = (Prescription dose) * (1+ (Dose per fraction/α/β)).

### Other intracranial treatment

Patients could not have undergone other intracranial therapy such as whole brain radiation therapy prior to this course of SRS. Patients were included regardless of receipt of previous systemic therapy or surgery. If patients were treated with surgery to an intracranial lesion, then the SRS treatment targeted the intracranial operative bed.

### Data collection and statistical analysis

Patients were followed clinically by their treating radiation oncologist, neurosurgeon and/or medical oncologist after treatment. Radiographic follow-up was completed with contrast-enhanced MRI and was read by a specialist in neuroradiology. Typically, first radiographic follow-up occurred 1-2 months after treatment, with additional follow-up MRIs performed every 6 months, or sooner if patients presented with neurologic symptoms. Radiographic follow-up time was determined by the date on which progression was found or the last study that demonstrated no progression. Follow-up brain PET/CT imaging was used at the discretion of the treating physician, typically to differentiate between recurrence and radionecrosis.

Treatment response was evaluated according to the standard Response Evaluation Criteria in Solid Tumors (RECIST). LC was defined as no tumor growth after treatment. DIF was defined as the absence of any new intracranial lesions after treatment. LC, DIF, and OS were estimated from the date of the first fraction of SRS.

Actuarial OS, LC, and DIF were calculated using the Kaplan-Meier method. Patients were censored at the time of the measured event or at the time of last clinical follow-up. The social security death index was used to corroborate date of death. Univariate analyses were performed using the logrank test, and multivariate analyses were performed using Cox proportional-hazards regression.

Toxicities were evaluated according to the Common Terminology Criteria for Adverse Events (CTCAE), Version 4.0, with specific attention to rates of intracranial edema and radionecrosis. Determination of radionecrosis was made through collaboration of the treating radiation oncologist, neurosurgeon, neuroradiologist, and clinical findings, when appropriate.

## Results

### Patient and lesion characteristics

Detailed patient characteristics are presented in Table 1. A total of 88 patients were treated, 48 (55%) at Georgetown, and 40 at UNC (45%). The majority of the patients were male (71%), with a median age of 65. Patients generally had good performance status, with a median ECOG score of 1 (Table 1). All patients had systemic disease, equally divided among those with stable (43%) or actively progressing (43%) disease. Twelve patients had systemic disease, but its status as stable or progressive was unknown. The vast majority of patients (78%) had additional sites of metastatic disease apart from the brain, and most patients were RPA class II, with only 4 patients in RPA class I, and 9 patients with unknown RPA status. Sixty-eight percent of patients were DS-GPA groups 1.5-2.5.

**Table 1:** Patient characteristics

**Characteristic**	***n *(%)**
**Total patients**	88
**Site of treatment**	
Georgetown University Hospital	48 (55%)
University of North Carolina	40 (45%)
**Sex** (***n***) (**per patient**)	
M	71 (81%)
F	17 (19%)
**Age** (**y**) (**per patient**)	
Median	65
Range	37-84
**ECOG Performance Status**	
Median	1
0	26 (30%)
1	30 (34%)
2	18 (20%)
3	4 (5%)
4	2 (2%)
Unknown	8 (9%)
Status of systemic disease*	
Stable	38 (43%)
Progressive	38 (43%)
Unknown	12 (14%)
Extracranial metastases**	
Present	69 (78%)
Absent	16 (18%)
Unknown	3 (3%)
**Recursive Partitioning Analysis Class**	
1	4 (5%)
2	67 (76%)
3	8 (9%)
Unknown	9 (10%)

* Systemic disease present, unknown to be stable vs. progressive, in 12 patients.

** Status unknown in 3 patients.

Variables relating to the intracranial lesions are presented in Table 2. Notably, only 6% of patients underwent surgery prior to SRS, and 47% of patients had received at least one dose of chemotherapy prior to SRS treatment. Lesions overall were small, with a median size of 13 mm (range, 1.5-56 mm), as determined by MRI. Dosimetric parameters, presented in Table 3, demonstrated the various doses and fractionation schema used for treatment. Although all patients were treated using the CyberKnife system, some patients were treated in a single fraction, while others were treated in hypofractionated treatment plans of 2–5 fractions. The BED varied substantially, with a median BED_10_ of 60 (range, 28–81.6).

**Table 2:** Lesion characteristics

**Characteristic**	***n *(%)**
**Location of lesion**	
Frontal	24
Parietal	19
Temporal	10
Occipital	11
Cerebellar	20
Other	4
**Surgery prior to SRS**	5 (6%)
**Chemotherapy prior to SRS**	41 (47%)
**Radiographic size** (**MRI**, **mm**)	
Median	13
Range	1.5-56

**Table 3:** Dosimetric parameters

**Characteristic**	***n***
**Prescribed dose**, **Gy**	
Mean	22.6
Median	20
Range	18-40
**Number of fractions**	
Median	1
Range	1-5
Median dose per fraction	2000
**BED**_**10**_, **Gy**	
Mean	55.7
Median	60
Range	28-81.6
**Prescription isodose line**, %	
Mean	81
Median	80
Range	70-92

### Clinical outcomes

Overall survival (OS), local control (LC) and distant intracranial failure (DIF) are summarized in Table 4. Of patients alive at last follow-up, median follow-up time was 11.1 months; median OS for the entire cohort was 10.6 months. One-year actuarial survival was 47.9%. Patients had excellent 1-year actuarial LC of 89.1%, and 1-year actuarial DIF of 61.2%.

**Table 4:** Clinical outcomes

**Endpoint**	**Time (months)**
Median follow-up, living patients	11.1
Median survival	10.6
Median local control	41.5
**Distant Intracranial Failure**	
Median DIF, all patients	17.3
Median DIF, progressive disease	6.2
Median DIF, stable disease	Not reached
**1**-**year outcomes**	% **Survival**
1-year actuarial OS	47.9%
1-year actuarial LC	89.1%
1-year actuarial DIF	61.2%

**Figure 1:**
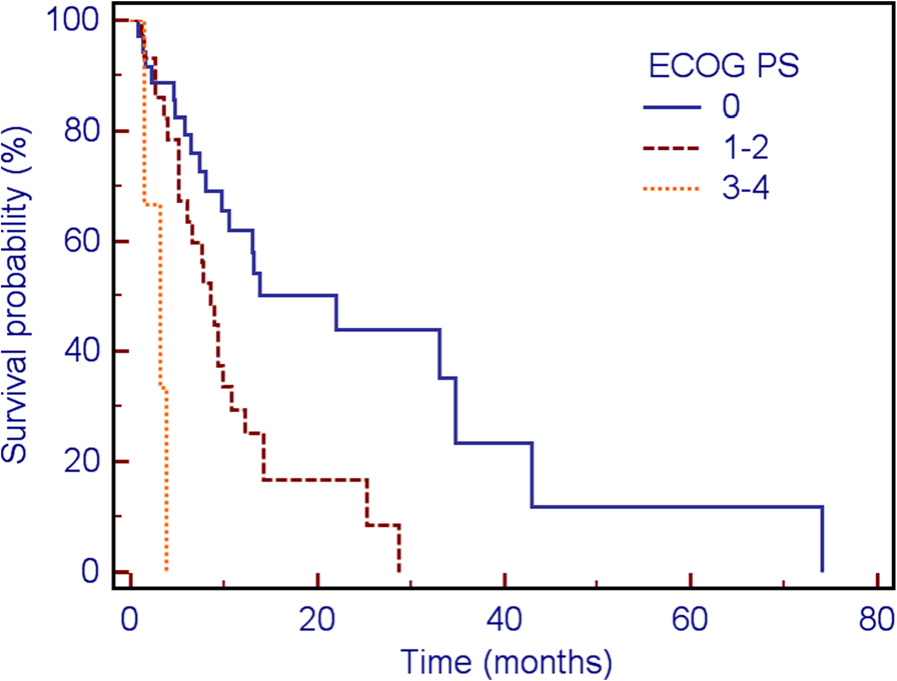
Impact of ECOG performance status on survival.

On univariate analysis (UVA), ECOG performance status (p = 0.001) and RPA class (p = 0.017) were associated with overall survival, but DS-GPA was not (p = 0.32). When patients were placed into three groups: ECOG = 0; ECOG = 1-2; ECOG = 3-4, the association persisted (p = 0.001, Figure 1). On multivariate analysis (MVA), only ECOG persisted as a significant factor (p = 0.036 when modeled with RPA class; p = 0.0081 when modeled without RPA class). No factors were found to be associated with LC on either UVA or MVA.

**Figure 2:**
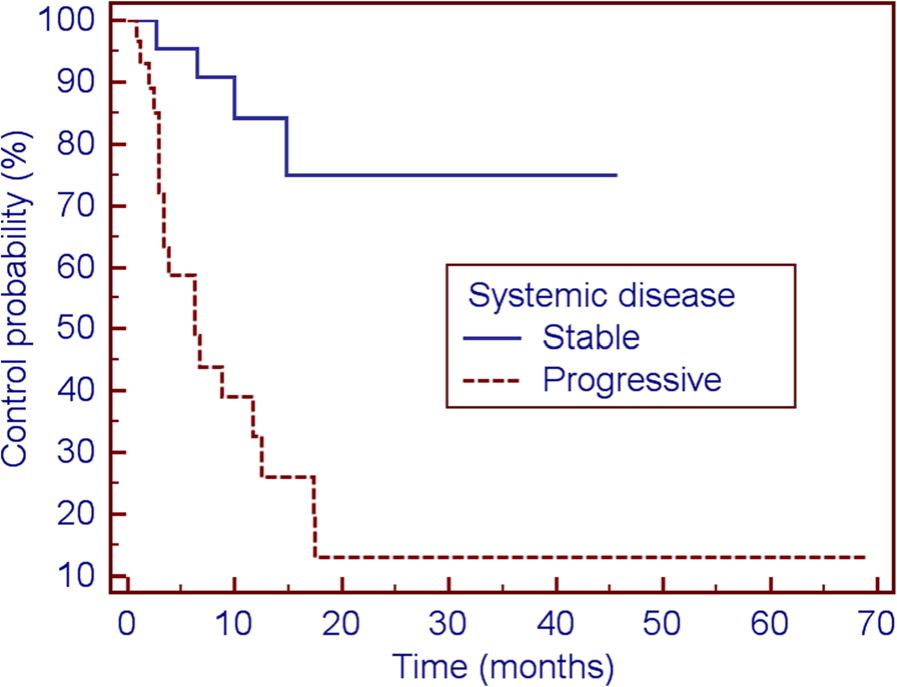
Impact of systemic disease status on distant intracranial failure.

On UVA, both BED (p = 0.021) and systemic disease status (p = 0.001, Figure 2) were found to be associated with DIF; only systemic disease status (p = 0.001) persisted as a significant factor on MVA (Table 5). Using the Wilcoxon rank-sum test, systemic disease status was not associated with radiographic follow-up time (*p* = 0.2701). A post-hoc power calculation to estimate the sensitivity to detect a difference in DIF based on systemic disease status was performed and predicted 99% power to detect type I/II error of 0.05. A total of 11 patients (13%) underwent a salvage course of RT, either to the previously treated lesion, a separate lesion within the brain, or a course of WBRT.

**Table 5:** Multivariate analysis of distant intracranial failure

**Variable**	**p**-**value**	**Exp (b)**	**95% CI of Exp (b)**
Systemic disease status	0.0008	9.46	2.54 – 35.14
Treatment Site	0.93	1.05	0.35 – 3.19
BED	0.13	0.31	0.069 – 1.38
Size	0.43	1.72	0.45 – 6.52
Surgery	0.19	3.51	0.53 – 23.16
Age	0.76	0.87	0.34 – 2.19
Extracranial metastases	0.49	0.59	0.13 – 2.62
ECOG Performance Status	0.59	1.18	0.64 – 2.18
Local Control	0.61	0.54	0.05 – 5.69

### Toxicity

Toxicities were minimal. In the acute setting, four patients experienced radionecrosis, two had intracranial edema, two developed mild fatigue, and one developed mild headache. None of the acute toxicities required intervention. The four patients with acute radionecrosis were treated in a range of 1-3 fractions and had tumors ranging 13-23 mm in size. One additional patient developed late radionecrosis. Overall, a total of five patients developed radionecrosis at any time point (6%). The majority of cases of radiation necrosis were asymptomatic and were found only on radiographic imaging. Symptomatic radiation necrosis was treated with oral steroids. No patient required craniotomy for refractory radiation necrosis.

## Discussion

Our study’s novel finding is that the status of extracranial disease at the time of SRS treatment predicts more strongly than any other clinical or demographic variable for DIF. As a result, it may be possible to more effectively and prospectively identify patients who are most or least likely to have their intracranial disease entirely controlled by SRS alone. This criterion could significantly impact patient care by optimally selecting patients for SRS alone, while limiting the likelihood of needing salvage therapy.

Stereotactic radiosurgery (SRS) is increasingly being used for patients with brain metastases. Debate continues as to whether SRS is an appropriate first-line treatment without the additional use of surgery or whole brain radiation therapy (WBRT), and which endpoints, including OS, LC, DIF, and QOL are the most significant in this patient population [16,22]. Limited data have demonstrated that in selected patients, an initial approach that includes SRS alone is appropriate [11,16]. However, these data are tempered by the fact that in clinical practice, many patients do not fit these trial criteria, including those of advanced age, with progressive extracranial disease, and/or poor performance status. As a result, many gaps remain in the literature regarding patient outcomes when using SRS alone as an initial approach. At the same time, the enthusiasm for embarking on SRS alone as initial therapy is necessarily tempered by the risks of development of distant intracranial disease, including risks of neurocognitive decline related to disease progression [14,23]. Although patient-reported outcomes (PROs)/QOL have not been studied among many subgroups of patients with brain metastases, it is also possible that an initial approach with SRS alone may lead to improved PROs/QOL for some who avoid or asymptomatically delay further intracranial treatment, while impaired long-term PROs/QOL for others who experience recurrence and require salvage therapy in the setting of pre-existing increase in symptoms. As a result, identification of factors predictive of distant intracranial recurrence is essential to refining patient selection for SRS, WBRT, or combination therapy.

Predicting survival of patients with brain metastases also has clinical utility. The first historical system developed for estimating patient prognosis, primarily during an era where treatment included WBRT and/or surgery, were the Recursive Partitioning Analysis (RPA) classes [5]. These classes, separating patients based upon age, status of primary tumor, and performance status, created rough prognostic groupings that, while useful, left large, heterogeneous groups of patients within the same category. For example, in our study, 76% of patients were considered RPA class II. A more modern system, the Diagnosis-Specific Graded Prognostic Index (DS-GPA), incorporates age, KPS, presence of extracranial metastases, and number of brain metastases to develop a score predictive of survival [6]. However, in our series, the DS-GPA was not found to be significantly associated with survival, suggesting that there still exists significant heterogeneity, even among patients within similar prognostic groups. Even more interestingly, the majority (68%) of the patients in this study had DS-GPA scores between 1.5-2.5, which would result in a projected median survival of 6.53 months. Instead, we have demonstrated prolonged survival of over 10 months in this carefully selected patient group, which underscores the importance of considering patients’ longer-term quality of life if they have limited brain metastases, even in the setting of additional adverse prognostic factors.

Although the present study has significant findings regarding survival and DIF, it does have several limitations due to its retrospective nature. Radiographic follow-up did not occur at consistent intervals for the entire study population, so events relating to LC and DIF may have been discovered, in some patients, with lag-time between development of disease and its recognition. The sample size of this study is still relatively small, and all power calculations were completed post-hoc due to the retrospective data collection and analysis. Additionally, no prospective quality of life measures or patient-reported outcomes were included in this study, which are of paramount importance in the palliative setting. Finally, patients were permitted to have additional systemic or local therapies at the discretion of their physicians, and preceding, concurrent, or adjuvant chemotherapy may have impacted DIF and OS in ways that were not analyzed in this study.

## Conclusions

Patients with NSCLC with a single brain metastasis in the setting of residual extracranial disease live longer than might be predicted by traditional prognostic algorithms, including RPA class and DS-GPA. During their prolonged survival, both LC and DIF are remarkably high. The sole factor that increased the risk of distant intracranial failure was progressive systemic disease at the time of SRS. Prospective studies are needed to optimize patient selection for up-front SRS, and to refine follow-up schedules that minimize the impact of distant intracranial failure on patients’ quality of life.

## Competing interest

Brian Collins, MD is a paid speaker for Accuray. The remaining authors have no competing interests.

## Authors’ contributions

This project was not supported by outside funding. MSK, EO, MGE, and BC all contributed to study concept and design. MSK, EO, and HC completed the data collection. MSK, EO, and RBH contributed to the data analysis. MSK contributed to drafting the manuscript. All authors contributing revising and giving final approval to the manuscript.

## Disclosures

Brian Collins, MD is a paid speaker for Accuray. The remaining authors have no financial disclosures.

## Meeting presentation

This project was presented at ASTRO’s 2012 annual meeting.
